# Superior biomechanical stability of pedicle screws compared to lateral mass screws: recommendations for bicortical positioning and enhancing bone contact in geriatric C1 vertebrae

**DOI:** 10.1186/s13018-025-05472-1

**Published:** 2025-01-18

**Authors:** Leon-Gordian Leonhardt, Leonie Rörup, Anna Lena Kammal, Michael Hahn, Marc Dreimann, Benjamin Ondruschka, Felix Nikolai von Brackel, Tim Rolvien, Lennart Viezens, Simon von Kroge

**Affiliations:** 1 Department of Trauma and Orthopaedic Surgery, University Medical Center Hamburg- Eppendorf, Martinistraße 52, 20246 Hamburg, Germany; 2 Institute of Legal Medicine, University Medical Center Hamburg-Eppendorf, Butenfeld 34, 22529 Hamburg, Germany; 3 Department of Osteology and Biomechanics, University Medical Center Hamburg-Eppendorf, Lottestraße 59, 22529 Hamburg, Germany

**Keywords:** Cervical spine surgery, Lateral mass screw, Pedicle screw, Mechanical testing, C1, Bone microstructure

## Abstract

**Background:**

In atlantoaxial instabilities, posterior C1/C2 fusion using lateral mass screws (LMS) or pedicle screws (PS) in a mono- or bicortical position in the atlas is a typical treatment. The bone microstructure and positioning of the screw trajectories appear to be of significant relevance for stability.

**Purpose:**

The aim of this study was a comparative analysis of the mechanical durability of screw fixation concerning microstructural characteristics of the trajectories of LMS and PS in mono- and bicortical position.

**Methods:**

Human C1 from geriatric body donors (*n* = 28; 50% female, age 80.8 ± 13.9 years) were collected and characterized based on their bone microstructure. Additionally, the mechanical stability of LMS and PS fixation in mono- and bicortical positioning was tested by mechanical loading. High-resolution quantitative computed tomography was used to analyze the bone microstructure of cylinders corresponding to the trajectories of PS and LMS in mono- and bicortical locations in each C1. After instrumentation with both screw types and types of fixation, the mechanical stability was tested by increased cyclic loading in cranio-caudal direction.

**Results:**

Trajectories of PS presented with more bone volume and a higher contact length to cortical bone. Simultaneously, a higher number of cycles and a higher maximum force was needed to loosen PS compared to LMS, while the loose by torque at the experiment end was still greater in PS. Differences between mono- and bicortical positioning of PS and LMS have only been observed in the initial stiffness of screws. When comparing microstructural and mechanical properties, the cortical contact length and bone volume in screw trajectories were strongest associated with a high loose and cycle count.

**Conclusions:**

This study suggests that mono- and bicortical positioning of PS is similarly efficient in creating a stable basis for screw fixation in the atlas. While PS are superior to LMS, the contact with cortical bone is of major relevance for a stable foundation.

**Supplementary Information:**

The online version contains supplementary material available at 10.1186/s13018-025-05472-1.

## Introduction

Atlantoaxial instabilities (AAI) are often occurring in geriatric patients even after low-energy trauma such as falls [1–3]. Due to an aging population in the majority of countries, the incidence of Dens axis fractures inevitably rises [4–6]. Posterior screw fixation of C1/C2 using screws and rod systems is the gold standard for treating AAIs [7–9]. The Placement of C1 screws can mainly be performed in two different ways: Harms et al. proposed a screw fixation inserted directly into the lateral mass (LMS) [8]. The alternative technique for screw fixation is dorsally entering the C1 arch, i.e., the pedicle, and reaching into the lateral mass of the C1 [9, 10]. Although both techniques provide adequate stabilization, the preferred choice of posterior screw fixation is still under discussion.

Several biomechanical studies have already analyzed the properties of the two types of posterior fixation. It has been shown that screws inserted in the pedicle of C1 (PS) present stiffer than LMS and that monocortical PS have the same pull-out strength as bicortical LMS [11]. Furthermore, PS in monocortical position are assumed to be biomechanically superior to LMS when applying craniocaudal pressure and tensile forces [12].

Although bicortical LMS in C1 show higher pullout strength than monocortical LMS, the necessity of using bicortical C1 screws in clinical practice is questioned in the literature [13]. One reason is the higher pullout strength of monocortical LMS in C1 than subaxial LMS. It is argued that the risk of injury to neurovascular structures ventral to C1 appears to be greater than the benefit of a bicortical screw position [13]. However, especially in geriatric patients with impaired bone quality and microstructure, bicortical positioning of screws could provide a clinically relevant advantage over monocortical screws, which could potentially lead to a clinical paradigm shift.

To date, there are no studies investigating the influence of a bicortical position of C1 PS on its biomechanical properties in association with the bone microstructure in a geriatric cohort.

Furthermore, the application of craniocaudal pressure and tensile forces appears to be more relevant for the loosening of screws in C1 than the pullout strength [12]. Hence, the objective of this study was to characterize the mechanical properties of relevant surgical techniques in the field of AAI treatment in geriatric patients, applying craniocaudal pressure and tensile forces. Specifically, we aimed to determine whether bicortical fixation is superior to monocortical fixation in screw trajectories through the atlas pedicle and with LMS. Moreover, we aimed to identify the microstructural determinants which are associated to the screw stability.

## Materials and methods

To characterize the mechanical properties of mono- and bicortical implantation of LMS and PS in C1, human atlases were collected, scanned, instrumented, and tested in a quasi-dynamic testing regimen. To specify the determinants of mechanical properties, the bone microstructure was assessed by high-resolution quantitative computed tomography. This study was reported to the local ethics committee (2022-300148-WF) and complied with the Declaration of Helsinki.

### Sample acquisition and preparation

In total, *n* = 28 atlases (50% female, age 80.8 ± 13.9 years) were sampled post-mortem by the Institute of Legal Medicine (University Medical Center Hamburg-Eppendorf). After approval of next of kin, the C1 to C3 of body donors were carefully removed during autopsy, separated, and stripped off residual soft tissue. Each atlas was directly frozen at -20 °C until further preparation. Clinical data was available for all individuals. Patients with previous or current fractures in the cervical spine, a history of surgeries in the cervical spine or tumor diseases were excluded from sampling. All samples were anonymized prior to further analysis.

### High-resolution quantitative computed tomography

The bone microstructure within and surrounding the trajectories of both LMS and PS was determined to correlate mechanical properties to structural aspects of LMS and PS. Prior to instrumentation, all atlases were scanned in total using a XTremeCT II (Scanco Medical AG, Brüttisellen, Switzerland). Scanning was performed with an acceleration voltage of 68 kVp, an anode current of 1470 µA, an integration time of 43 ms, and a voxel size of 30.3 μm. A volume of interest (VOI) ranging from the dorsal entry point of each trajectory to the ventral egress point with specific diameters was analyzed. For exact location, landmarks of clinical routine and previously determined safe zones for screw placement were used [14]. In detail, diameters of 1.8 mm, 3.5 mm, and 4.0 mm were analyzed, representing the drill canal, screw diameter, and surrounding tissue of screws, respectively. Additionally, to determine the microstructure in trajectories of monocortical fixation, 64 slides, i.e., 1.94 mm, were subtracted from the evaluation. In total, the microstructure and mineralization of each VOI were assessed in all 28 specimens. More specifically, the bone volume (BV, mm^3^) and volumetric bone mineral density (vBMD, mg HA/mm^3^) of all VOIs, the total insertion length of the screws (Tt.Length, mm), the cortical contact length (Ct.Length, mm), and based on the bone volume fraction the estimated trabecular contact length (Tb.Length, mm) of screw trajectories were determined.

### Sample instrumentation

**Fig. 1:**
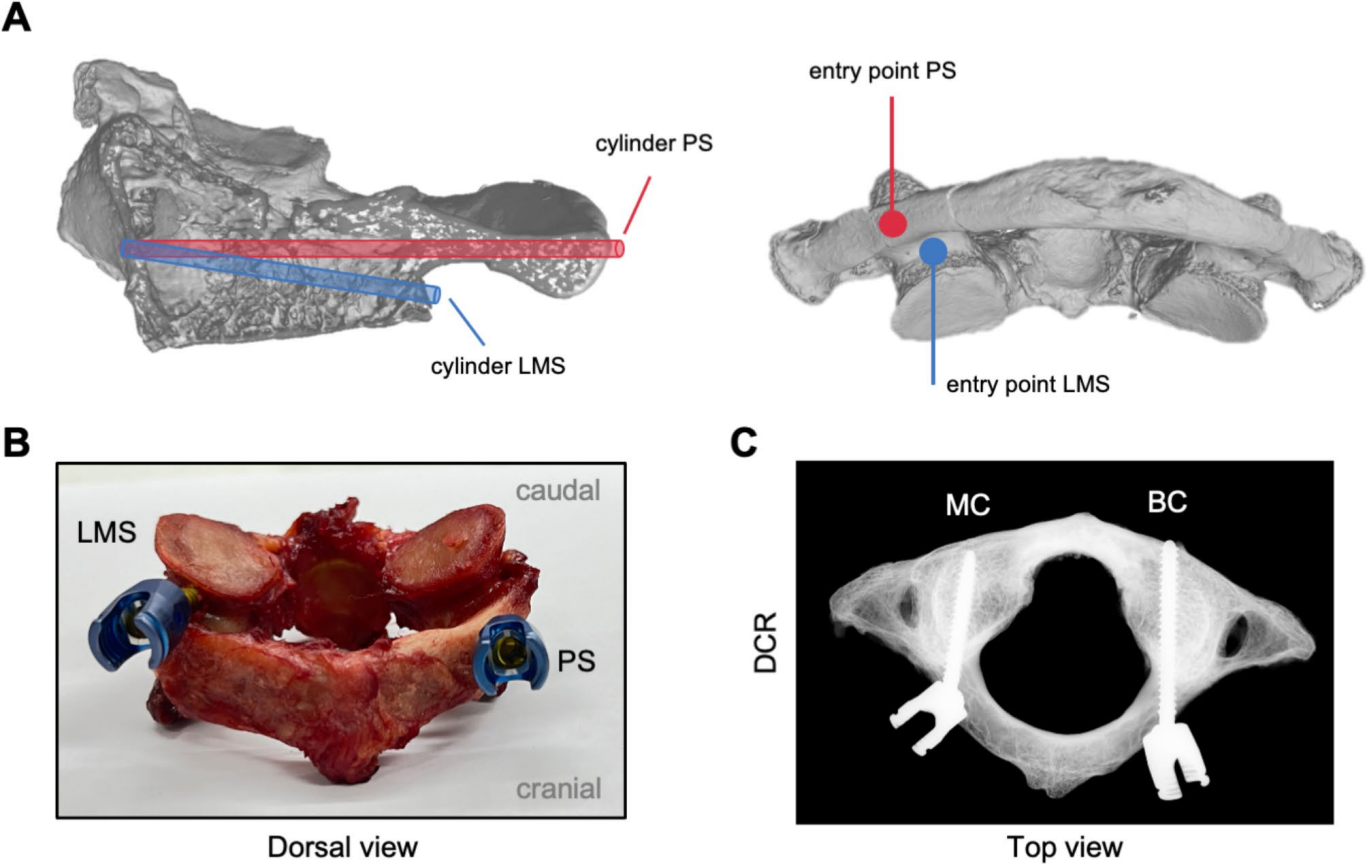
Representative scheme of an atlas and imaging of screw trajectories in mono- or bicortical screw fixation. (**A**) Three-dimensional renderings of an atlas with indicated cylindric trajectories of pedicle screws (PS) in red and lateral mass screws (LMS) in blue, as well as their entry points at the dorsal side of an atlas (right side). (**B**) Dorsal view of a representative atlas instrumented with a lateral mass screw on the left and a pedicle screw on the right. (**C**) Top view of an atlas obtained by digital contact radiography (DCR) illustrating monocortical (MC) and bicortical (BC) instrumentation.

The instrumentation of all C1 was conducted by one trained spine surgeon. Instruments were kindly provided by, and screws were purchased from DePuy Synthes (DePuy Spine Inc., MA, USA). Detailed information on used screws is listed in the Supplementary Material. Based on demographic data, C1 were matched and allocated to four groups, i.e., monocortical PS, bicortical PS, monocortical LMS, and bicortical LMS (Table 1). Each atlas was instrumented with one PS and one LMS on the other lateral side, hence, *n* = 14 mechanical measurements were performed for each type of fixation, i.e., mono- and bicortical LMS and mono- and bicortical PS. Like VOIs in high-resolution quantitative computed tomography, the positioning of screw trajectories was based on clinical routine and previously determined safe zones for the placement of PS in the atlas (Fig. 1A) [14]. In brief, the sample instrumentation was performed by drilling a canal of 1.8 mm in diameter, cutting a thread with a diameter of 3.5 mm (screw diameter), followed by screw insertion. The correct position of LMS and PS was afterwards visually checked and confirmed by digital contact radiography (DCR) (Fig. 1B, C).

**Table 1:** Overview of characteristic data from samples that were allocated for mechanical testing

	Overall	LMS		PS		*p-value*
		Bicortical	Monocortical	Bicortical	Monocortical	
Sex (male/female)	14 / 14	8 / 6	6 / 8	8 / 6	6 / 8	0.767
Age (years)	80.8 (13.9)	79.1 (15.8)	82.3 (12.1)	80.0 (11.9)	81.6 (16.0)	0.828
Height (cm)	170 (10)	169 (10)	171 (10)	171 (11)	168 (9)	0.696
BMI (kg/m^2^)	24.8 (7.2)	25.9 (5.6)	23.9 (8.5)	25.6 (7.9)	24.4 (6.5)	0.838
BV_total_ (mm^3^)	71.0 (30.4)	57.3 (16.4)	43.8 (14.9)	97.7 (23.4)	85.1 (29.7)	< 0.001
vBMD (mgHA/mm^3^)	284 (122)	229 (83)	240 (101)	334 (114)	333 (149)	0.03
Tt.Length (mm)	22.3 (4.6)	21.1 (2.1)	16.8 (1.8)	28.0 (3.0)	23.4 (2.1)	< 0.001
Ct.Length (mm)	5.5 (3.4)	4.3 (1.7)	2.4 (1.1)	8.7 (2.4)	6.5 (3.9)	< 0.001
Tb.Length (mm)	5.7 (1.9)	4.8 (1.0)	4.3 (1.4)	7.6 (1.5)	6.2 (1.4)	< 0.001

Data is presented as mean (standard deviation). Statistical testing for differences was performed by one-way ANOVA

LMS: lateral mass screw, PS: pedicle screw, BMI: body mass index, BV_total_: total bone volume, vBMD: apparent volumetric bone mineral density, Tt.Length: total insertion length, Ct.Length: cortical contact length, Tb.Length: estimated trabecular contact length

### Biomechanical testing

**Fig. 2:**
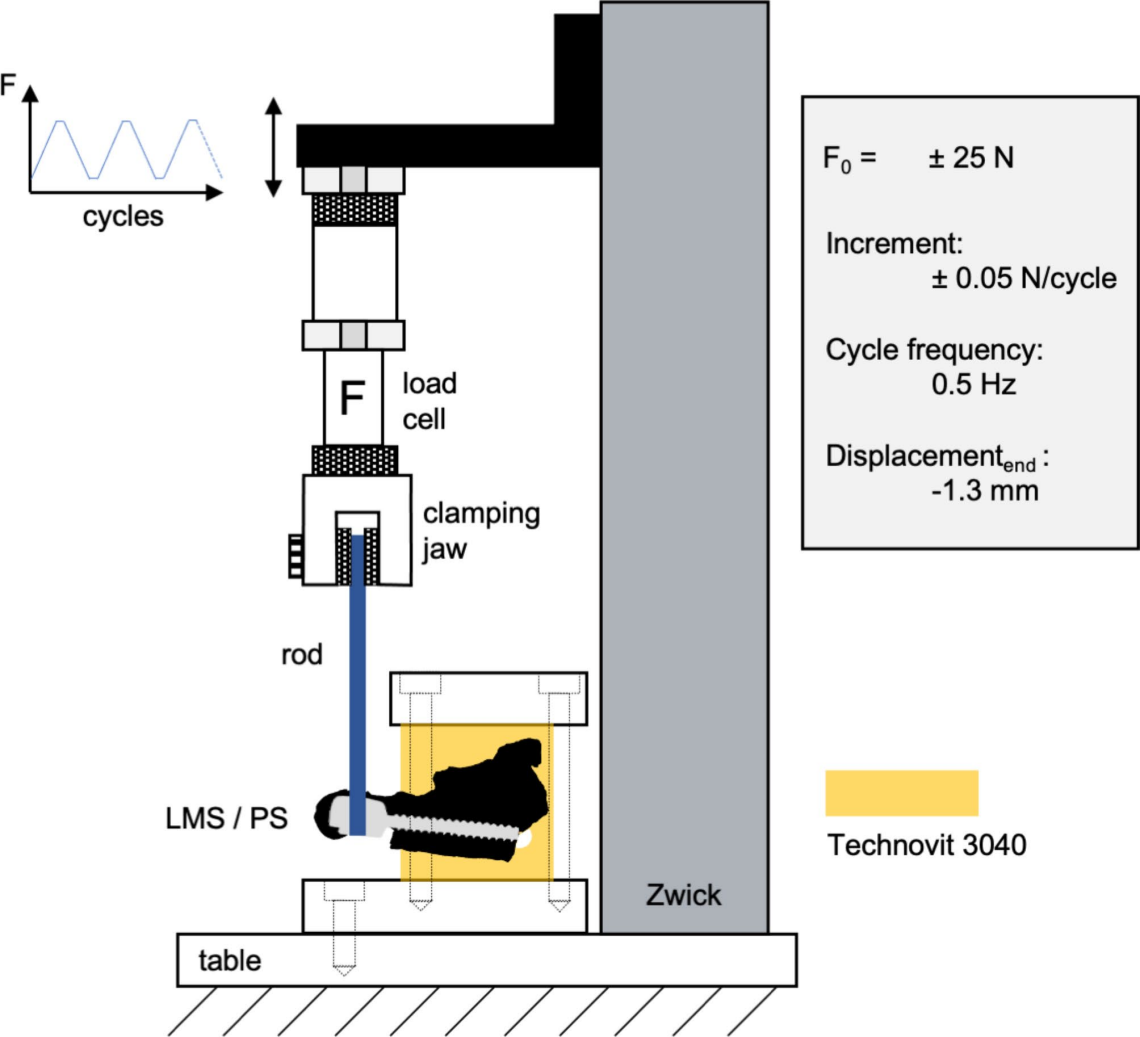
Scheme of the mechanical testing setup of LMS and PS fixation in atlases. Atlases were embedded in Technovit 3040 (yellow) and fixated by clamps attached to the testing machine’s table. The screw heads (LMS & PS) were stabilized with the corresponding metal rod template and grub screw. The metal pin was attached to a load cell by a clamping jaw and mechanically loaded by cyclic application of increasing force (F = -25 *N* ± 0.05 N/cycle). LMS: lateral mass screw, PS: pedicle screw, F_0_: initial loading

To determine the mechanical stability of the inserted screws within the atlas, loading was applied based on a previously published protocol [12]. Atlases were positioned in alumina cups, which facilitated as scaffolds, with a dimension of 100 × 25 × 25 mm^3^ and embedded in Technovit 3040^®^ so that the resin surface reached 1 mm below the screw entry points. Using dental wax (Ortho Technology, West Columbia, USA) the egress points were kept free of resin. The atlas-resin blocks were detached from alumina scaffolds and clamped in a custom-made mounting ensuring fixation at the base of the testing machine and coupled with the load cell *via* the rod template by the screw manufacturer (Fig. 2). Mechanical testing was performed using a ZwickiLine Z2.5 (ZwickRoell GmbH & Co. KG, Ulm, Germany) testing machine. During fixation, mounting, and testing, atlases were kept moist using bandages soaked with 0.8% sodium chloride solutions. The machine was operated with a sinusoidal loading implying alternating loading in compression and tension starting at F_0_ of ± 25 N, an increment of 0.05 N per cycle, and 0.5 Hz for each cycle. Testing ended at 1.3 mm total displacement. Subsequently, the atlas-resin block was detached from the testing machine and the loose torque was measured. For comparison, the initial displacement (at cycle no. 1), the initial and end stiffness, the number of cycles, and the maximum force applied were determined. The instrumentation and biomechanical testing procedure was first tested on *n* = 5 artificial C1 (Sawbones, Pacific Research Laboratories, Inc., Vashon, USA) instrumented with both mono- and bicortically inserted PS and LMS.

### Statistical analysis

The estimated sample size calculation was performed using G*Power software (v. 3.1.9.2; Heinrich-Heine-Universität Düsseldorf, Düsseldorf, Germany). With a statistical power of 0.8, an alpha risk of 0.05, and an estimated effect size *f* of 0.955 (based on the previously published data by Fensky et al. [12]) a minimum total sample size of *n* = 20 was determined to perform a two-way ANOVA. A number of *n* = 28 C1 was selected for this study to prevent the effect of unplanned losses. Statistical analysis was performed using SPSS software (v 24.0, IBM, USA). Differences between the morphometry in VOIs and mechanical properties of screw fixation were determined by two-way ANOVA and subsequent post hoc (Tukey) testing. The type of fixation (mono- and bicortical) and screw type (LMS and PS) were selected as independent nominal variables. Data is presented as mean ± standard deviation. Correlation analyses were performed using the Pearson correlation coefficient or the Spearman r coefficient in case of a non-normal distribution. A significance level of 0.05 was chosen.

## Results

### Pedicle screw trajectories contain more cortical and trabecular bone

**Fig. 3:**
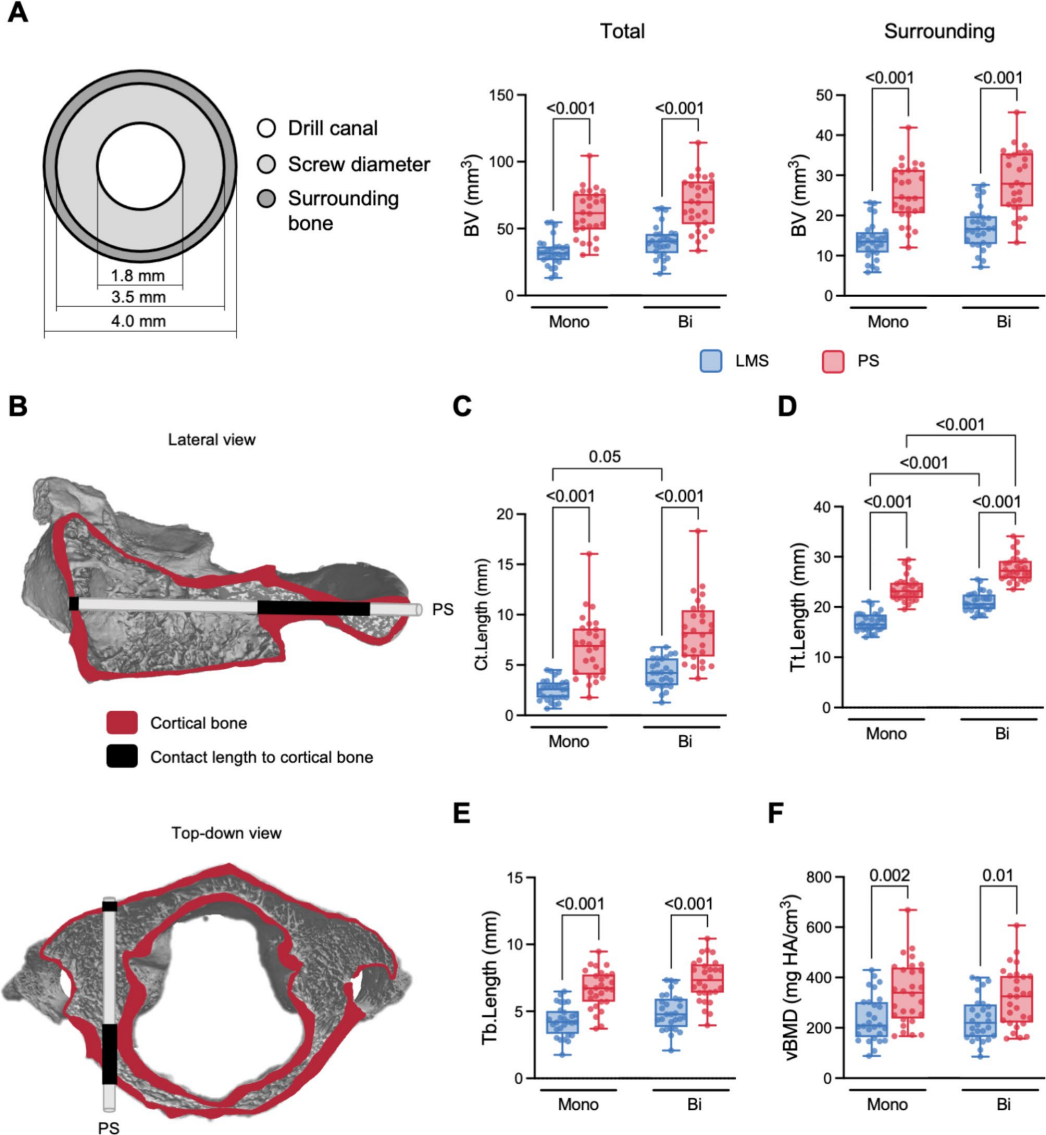
Microstructural characteristics of screw trajectories in the atlas. (**A**) A scheme illustrating the bone volumes measured by high-resolution quantitative computed tomography. In both the total (including the screw diameter and surrounding bone, and not the drill canal) and the surrounding bone volume, PS trajectories presented with a higher bone volume than LMS trajectories, while no difference between mono- and bicortical volumes were determined. (**B**) Representative lateral view and top-down view of a bicortical PS cylinder location to illustrate the cortical contact length passed (black). (**C**) PS presented with a longer cortical contact compared with LMS. Additionally, a trend towards a higher contact length in bicortical trajectories than in monocortical trajectories was apparent. (**D**) The total insertion length of the screws was higher in bicortical than in monocortical position, and in PS compared with LMS. (**E**) The estimated contact to the trabecular bone of PS was longer compared with LMS in both mono- and bicortical positions. (**F**) In PS a higher apparent volumetric bone mineral density (vBMD) than LMS was observed, while mono- and bicortical trajectories did not show differences

The bone volume and bone mineral density of LMS and PS trajectories were determined in the total (screw diameter without the drill canal & surrounding bone) and surrounding bone solely (Fig. 3A). Analyzing the effect of fixation, screw type, and their interaction, a significant dependency of screw type on bone microstructural parameters was determined, whereas no significant interaction of screw type and fixation was apparent (Table 2). More specifically, a statistical difference between bone volumes in monocortical and bicortical trajectories was not apparent in either LMS or PS. The total bone volume in the PS trajectories was approximately twice as high as in LMS trajectories (monocortical: 86.9 ± 24.6 mm^3^vs. 46.3 ± 15.0 mm^3^, *p* < 0.001; bicortical: 97.9 ± 27.4 mm^3^vs. 57.4 ± 17.6 mm^3^, *p* < 0.001). Similarly, the bone volume of the surrounding bone in the PS trajectories was approximately twice as high as in LMS trajectories (monocortical: 25.5 ± 7.0 mm^3^vs. 13.7 ± 4.6 mm^3^, *p* < 0.001; bicortical: 28.6 ± 7.8 mm^3^vs. 17.0 ± 5.3 mm^3^, *p* < 0.001). The cortical contact length (which is exemplarily shown in black in Fig. 3B) was higher in PS trajectories compared to LMS trajectories (monocortical: 6.7 ± 3.1 mm vs. 2.5 ± 1.1 mm, *p* < 0.001; bicortical: 8.4 ± 3.2 mm vs. 4.2 ± 1.5 mm, *p* < 0.001). Further, the cortical contact was longer in the bicortical compared to the monocortical volume of interest in LMS (*p* = 0.049) but not PS (*p* = 0.06) (Fig. 3C). As expected, the total insertion length of bicortically positioned screws (PS: 27.7 ± 2.5 mm vs. 23.5 ± 2.4 mm, *p* < 0.001; LMS: 21.0 ± 1.9 mm vs. 17.0 ± 1.8 mm, *p* < 0.001) and PS (monocortical: *p* < 0.001; bicortical: *p* < 0.001) were larger compared with monocortically positioned screws and LMS, respectively (Fig. 3D). The estimated trabecular contact length was larger only in PS (monocortical: 6.7 ± 1.7 mm vs. 4.3 ± 1.2 mm, *p* < 0.001; bicortical: 7.3 ± 1.6 mm^3^vs. 5.0 ± 1.3 mm^3^, *p* < 0.001) compared with LMS (Fig. 3E). In PS trajectories, the included vBMD was significantly higher than in LMS trajectories (monocortical: 340 ± 126 mg HA/mm^3^vs. 238 ± 93 mg HA/mm^3^, *p* = 0.002; bicortical: 321 ± 116 mg HA/mm^3^vs. 235 ± 88 mg HA/mm^3^, *p* = 0.01). A difference between monocortical and bicortical VOIs was not apparent (Fig. 3E).

**Table 2:** Characteristic values determined by biomechanical testing

	LMS		PS		Screw		Fixation		Interaction		
	Bicortical	Monocortical	Bicortical	Monocortical	*p*	$\:{\eta\:}_{p}^{2}$	*p*	$\:{\eta\:}_{p}^{2}$	*p*	$\:{\eta\:}_{p}^{2}$	
Loose_Torque_ (Nm)	0.159 (0.106)	0.034 (0.049)	0.588 (0.389)	0.683 (0.417)	**< 0.001**	**0.494**	0.9	< 0.001	0.1	0.042	
F_max_ (N)	-77.4 (17.5)	-65.5 (18.3)	-121 (32.5)	-110 (11.0)	**< 0.001**	**0.512**	0.05	0.070	0.7	0.003	
Displacement_initial_ (mm)	-0.328 (0.058)	-0.407 (0.076)	-0.223 (0.052)	-0.269 (0.043)	**< 0.001**	**0.413**	**0.01**	**0.113**	0.9	< 0.001	
Cycles (1)	1061 (357)	818 (367)	1979 (594)	1668 (237)	**< 0.001**	**0.535**	**0.02**	**0.102**	0.5	0.011	
Stiffness_initial_ (N/mm)	75.7 (15.7)	62.7 (14.7)	118 (32.5)	93.3 (14.5)	**< 0.001**	**0.431**	**0.003**	**0.162**	0.2	0.039	
Stiffness_end_ (N/mm)	59.5 (13.6)	49.1 (14.2)	93.9 (24.1)	84.1 (9.5)	**< 0.001**	**0.529**	**0.03**	**0.088**	0.7	0.003	
Stiffness_change_ (N/mm)	16.2 (8.9)	13.6 (8.3)	23.9 (23.0)	9.2 (11.3)	0.6	0.005	**0.03**	**0.086**	0.1	0.058	

Two-way ANOVA was used to test for influences of screw type, mono- or bicortical fixation, and their interaction. The *p*-value as well as the effect size are reported, statistically significant effects are highlighted in bold

LMS: lateral mass screw, PS: pedicle screw, F_max_: maximal force, Stiffness_inital_: stiffness at cycle no. 1, Stiffness_end_: stiffness at end of testing, Stiffness_change_: difference between Stiffness_inital_ and Stiffness_end_

### Biomechanical superiority of bicortical placement in both pedicle screws and LMS

**Fig. 4:**
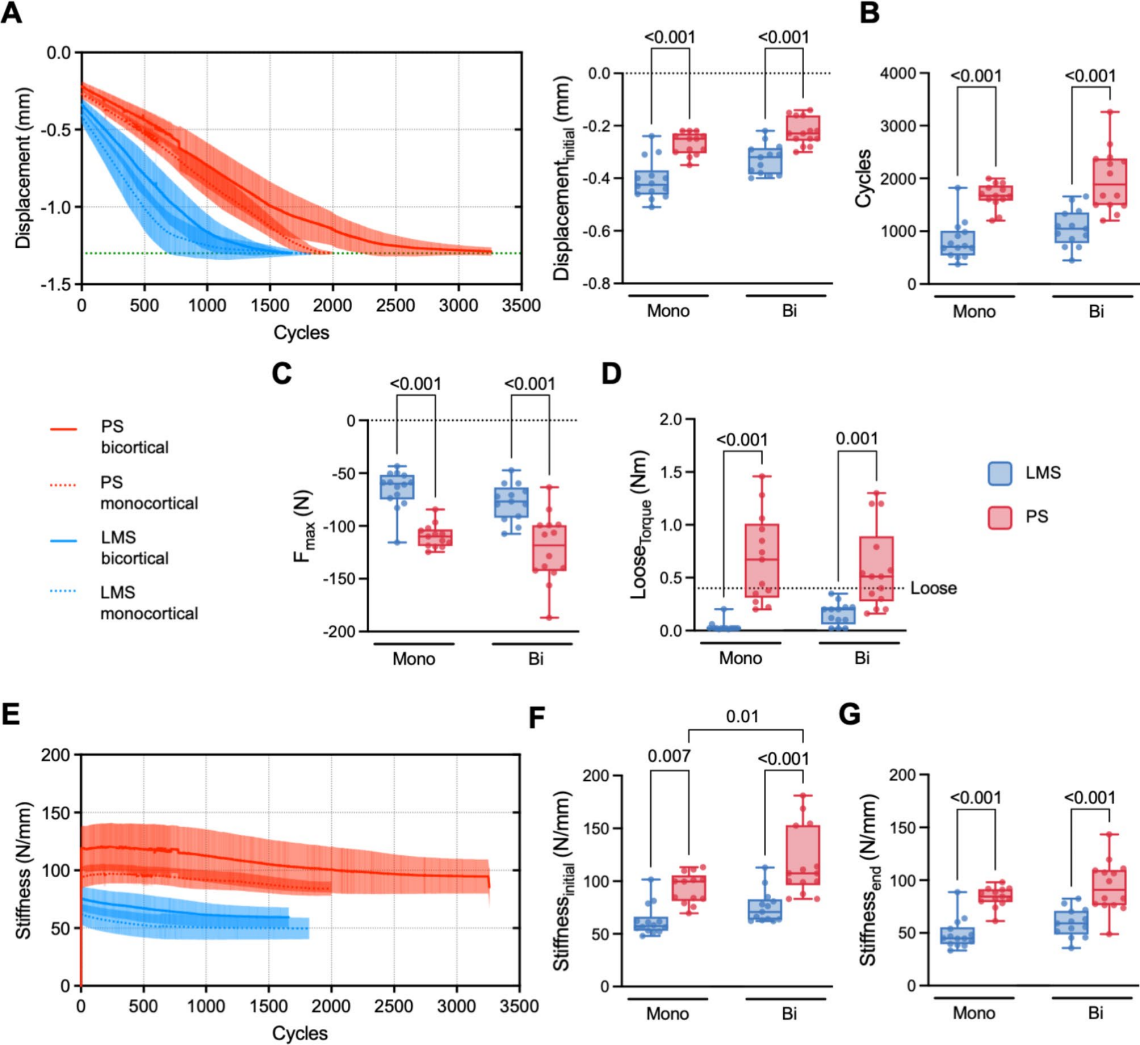
Mechanical properties of PS and LMS fixation. (**A**) The displacement during testing is presented over cycles indicating a different behavior predominantly between PS (red) and LMS (blue), while differences between mono- (pointed) and bicortical (straight) fixation were less pronounced. The green line indicates the test endpoint at -1.3 mm. The initial displacement of PS was higher than LMS, while no significant difference between bicortical fixation to monocortical fixation was observed. (**B**) PS fixation presented with more cycles until the test end than LMS fixation. (**C**) Likewise, the maximum force of PS fixation was higher than LMS fixation. (**D**) After loading, PS presented with an elevated loose (torque) indicating a higher end stability than LMS. (**E**) Similar to the displacement, the stiffness primarily differed between PS and LMS fixation. (**F**) In the initial phase, PS fixation presented stiffer than LMS fixation, and the bicortical placement of PS also presented stiffer than monocortical placement. (**G**) At the endpoint, the stiffness of PS was higher than that of LMS

The mechanical stability of PS and LMS in the monocortical and bicortical anchorage was assessed *ex situ* by cyclic loading in cranio-caudal direction. Similar to microstructural parameters, an interaction between screw type and fixation in mono- or bicortical anchorage was not determined for any measured mechanical characteristic. The displacement over cycles varied significantly between PS and LMS (Fig. 4A). While bicortical PS reached a displacement of -1.3 mm at the highest cycles, bicortical and monocortical LMS presented a steeper displacement during the full tests. The initial displacement (at test start) of PS was significantly smaller than LMS in both monocortical (-0.27 ± 0.04 mm vs. -0.41 ± 0.08 mm, *p* < 0.001, Cohen’s d = 1.573) and bicortical (-0.22 ± 0.05 mm vs. -0.33 ± 0.06 mm, *p* < 0.001, Cohen’s d = 1.660) fixation. Similarly, PS showed higher cycle counts until the test end than LMS in monocortical (1668 ± 237 mm vs. 818 ± 367 mm, *p* < 0.001, Cohen’s d = 1.870) and bicortical (1979 ± 594 mm vs. 1061 ± 357 mm, *p* < 0.001, Cohen’s d = 2.268) anchorage, while no differences between mono- and bicortical fixation was apparent (Fig. 4B). As the maximum force applied (F_max_) is directly associated with the cycle count, a larger F_max_ in both monocortical PS (-110 ± 11 N vs. -66 ± 18 N, *p* < 0.001, Cohen’s d=-1.867) and bicortical PS (-121 ± 33 N vs. -77 ± 18 N, *p* < 0.001, Cohen’s d=-2.081) was detected compared to LMS (Fig. 4C). After loading, the loose (torque) of each screw was determined. In both monocortical (0.68 ± 0.42 Nm vs. 0.03 ± 0.05 Nm, *p* < 0.001, Cohen’s d = 2.308) and bicortical (0.59 ± 0.39 Nm vs. 0.16 ± 0.11 Nm, *p* = 0.001, Cohen’s d = 1.501) anchorage PS presented with a higher loose than LMS. Although no significant difference between mono- and bicortical fixation was observed, most LMS in monocortical fixation presented nearly no remaining resistance against loosening. Additionally, no LMS reached a previously determined level of 0.4 Nm, representing stable fixation, while only 37% of PS failed to reach this level (Fig. 4D). The stiffness of all screws declined slowly and predominantly linearly during loading (Fig. 4E). Similar to the initial displacement, PS presented with a higher initial stiffness in monocortical (93.3 ± 14.5 N/mm vs. 62.7 ± 14.7 N/mm, *p* = 0.007, Cohen’s d = 1.289) and bicortical (118.0 ± 32.5 N/mm vs. 75.7 ± 15.7 N/mm, *p* < 0.001, Cohen’s d = 2.069) fixation (Fig. 4F). Further, the bicortical fixation showed a superior initial stiffness compared to the monocortical fixation of PS (*p* = 0.01, Cohen’s d = 1.239). This difference was not apparent in the final stiffness. Nevertheless, PS presented with a higher final stiffness compared to LMS in both monocortical (81.1 ± 9.5 N/mm vs. 49.1 ± 14.2 N/mm, *p* < 0.001, Cohen’s d = 1.942) and bicortical (93.9 ± 24.1 N/mm vs. 59.9 ± 13.6 N/mm, *p* < 0.001, Cohen’s d = 2.1434) fixation (Fig. 4G). The effect size of sex on all parameters that were evaluated was smaller compared with the type of screw positioning and fixation in each case (Suppl. Table 2).

### Biomechanical stability is directly associated with cortical/trabecular anchorage

**Fig. 5:**
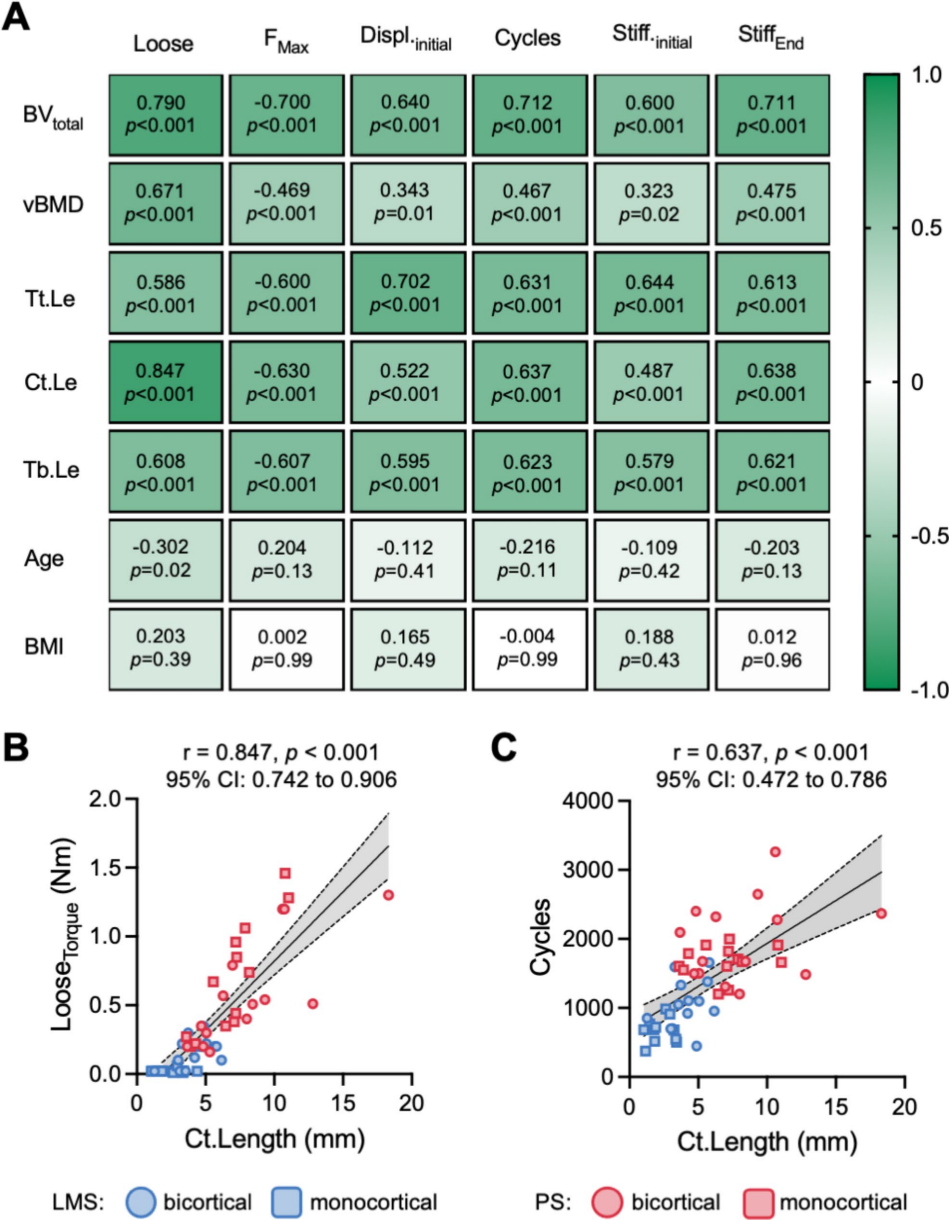
Correlation analyses of microstructural parameters and mechanical properties. (**A**) Heatmap of correlation coefficients reflecting associations between the screw trajectory microstructure (i.e., total bone volume, volumetric bone mineral density, total insertion length, cortical contact length, estimated trabecular contact length) and demographics (i.e., age and BMI of the individuals) with experimentally determined mechanical properties of LMS and PS. The r coefficient as well as p-values are depicted. The color coding reflects the r coefficient. (**B**) The loose by torque of LMS and PS at the end of the experiments correlated strongest with the cortical contact length, while PS in mono- and bicortical fixation presented with a higher cortical contact length and loose values. (**C**) Similarly, a strong correlation of cycle count with the cortical contact length was indicated, while PS in mono- and bicortical fixation presented with a higher cortical contact length and cycle counts

To derive determinants for screw fixation, a correlation analysis of microstructural and demographic data with mechanical properties was performed. Significant correlations were found between several parameters, while the surrounding bone volume and cortical contact length presented with highest correlation coefficients to nearly all mechanical properties (Fig. 5A). Interestingly, age showed only a low negative correlation (*r*=-0.302, 95% CI: -0.527 to -0.038, *p* = 0.03) with loose, while no correlations were found to other mechanical properties. Similarly, no correlations were found between BMI and mechanical properties of screw fixation (Fig. 5A). The cortical contact length correlated best with loose (*r* = 0.843, 95% CI: 0.742 to 0.906, *p* < 0.001). Interestingly, the bone volume, the bone volume fraction, and the cortical contact length showed stronger correlations with endpoint parameters, i.e., loose, F_max_, cycles, and final stiffness, than start parameters, i.e., initial displacement and stiffness. In contrast, the total insertion length and estimated trabecular contact length presented the highest correlation coefficients with parameters determining the mechanical stability at the experiment start, i.e., initial displacement and initial stiffness. When associating the cortical contact length with loose as a measure of stability, it was apparent that the association was independent of screw type and type of fixation (Fig. 5B). Similarly, the association of cycles as a measure of durability and cortical contact length showed no visible effect of screw type and type of fixation (Fig. 5C).

## Discussion

In this study, the mechanical properties of LMS and PS in atlases were analyzed to determine their stability in mono- and bicortical positioning and in dependency on the bone microstructure of their trajectories. We could show that the relevance of bicortical positioning is only minor with regard to craniocaudal loading and that surrounding bone volume as well as the cortical contact length are major determinants for successful stabilization.

While one previous study analyzed the mechanical properties of LMS and PS in terms of pull-out strength [11], this study focused on the cyclic application of craniocaudal compression and tensile forces like Fensky et al., who indicated that PS are more stable than LMS when craniocaudal loading is applied to the screw head [12]. Although rotation represents the main movement of C1/C2, a higher rate of dislocation after posterior fusion occurs in the sagittal plane due to extension and flexion. The authors attribute this to the pivot point located ventral to the posterior instrumentation, which results in large levers during extension and flexion [15]. Hence, we consider the application of craniocaudal loading on the screw head clinically more relevant.

A biomechanical superiority of PS to LMS due to possible contact of screws with bone has already been shown in the past [9, 14, 16–18]. This study has confirmed these findings by ex vivo experiments in cranio-caudal loading. In particular, PS presented with a higher loose torque than LMS and no LMS exceeded a previously described level of 0.4 Nm representing loosened screws [12, 19], while only 37% of PS did not exceed this level. Similarly, PS showed higher cycle counts and maximum force applied until the test end than LMS in mono- and bicortical anchorage. Additionally, we were now able to show that not only the bone volume traversed by the screw but particularly the length of cortical contact is larger in PS compared to LMS and that this is correlating with biomechanical data reflecting screw stability. The equivalent larger vBMD in PS compared to LMS trajectories has also already been associated with a higher stability of pedicle screws [20]. Although a direct cause-effect mechanism cannot be derived, the current study solidifies evidence that contact with cortical bone is of major relevance for the endurance of PS and LMS in treating AAI.

This study further illustrates that mechanical differences between mono- and bicortical anchorage are primarily present after insertion of screws. Specifically, the smaller initial displacement of PS and initial stiffness of LMS in monocortical positions were not reflected in mechanical properties measured at the end of testing. In pull-out tests, it has recently been indicated that bicortical positioning is superior to monocortical positioning of both LMS and PS [11, 13]. The authors concluded that a bicortical screw position could be recommended if there is an increased risk of material failure, especially when using LMS [11]. Such an increased risk is present, for example, in the clinically relevant geriatric collective of this study due to a higher risk of poor bone quality [21]. Although mechanical properties determined during this study do not fully affirm mono- and bicortical differences, the association of bone volume and cortical contact with mechanical properties supports the conclusion of preferred bicortical positioning in geriatric patients. To test for actual clinical superiority, a prospective controlled randomized trial would be needed.

### Clinical relevance

In total, how much bone volume traversed by screws and how much contact to cortical bone is present appear to be the most relevant factors for the stability of screws. Both should be maximized in clinical use to decrease the risk of loosening. As PS rather than LMS provide a higher chance of contact to trabecular and especially cortical bone, our data support an intervention preferably through the pedicle. By a bicortical positioning of the PS, the initial stiffness of screws can be increased, hence offering an advantage to monocortical positioning. Whether to use PS or LMS in mono- or bicortical position needs however to be allocated on an individual basis as anatomical conditions or high injury risks of surrounding soft tissue might rule out any other treatment.

### Strengths & limitations

This study provides the first biomechanical analysis of screw fixation of PS and LMS in mono- and bicortical positions in the geriatric human atlas, applying craniocaudal compression and tensile forces. By showing that rather bone volume and contact with cortical bone than bicortical position plays an overarching role against loosening, clinicians can easier be guided in selecting appropriate surgical interventions. However, this study is not free of limitations. Firstly, the experiments were performed *ex situ*, not considering soft tissue influences or force application in other directions than cranio-caudally. Secondly, the bone status before instrumentation of screws was assessed and compared with mechanical properties with installed PS and LMS. Although the direct analysis of bone contact with the screws would indicate mechanical properties more accurately, the assessment pre installation offers an evaluation closer to the clinical routine. Further, this study only comprises atlases of geriatric patients and is therefore not representative of other cohorts and age groups. Nevertheless, the chosen standardized experimental setup in mechanical testing as well as an analysis on the microstructural level is currently not applicable in vivo and provides the best approach to estimate loading in a clinical setup. Additionally, the dorsal intervention is primarily conducted in geriatric patients, which is why these experiments are representative of the most important patient cohort.

## Conclusions

Overall, fixation with PS appears to be biomechanically superior to fixation with LMS. Even though an advantage of bicortical positioning over monocortical positioning could only be shown regarding the initial stiffness of the PS, our data suggest that bicortical anchorage could be recommended in particular in geriatric patients. Traversed bone volume and the cortical contact length of screws should be maximized in clinical use to increase screw stability, as they correlate strongly with their biomechanical load-bearing capacity.

## Electronic supplementary material

Below is the link to the electronic supplementary material.

Supplementary Material 1

## Data Availability

The data generated and analyzed during the current study are available from the corresponding author upon reasonable request.
