# Supplementary material for: Superior biomechanical stability of pedicle screws compared to lateral mass screws: recommendations for bicortical positioning and enhancing bone contact in geriatric C1 vertebrae
Source: J Orthop Surg Res. 2025 Jan 18;20:63. doi: 10.1186/s13018-025-05472-1 (PMC11742196; doi:10.1186/s13018-025-05472-1)
Supplement: Supplementary file 1 — Supplementary Material 1 [file 13018_2025_5472_MOESM1_ESM.docx]

Supplementary Material

Superior Biomechanical Stability of Pedicle Screws Compared to Lateral Mass Screws: Recommendations for Bicortical Positioning and Enhancing Bone Contact in Geriatric C1 Vertebrae

Suppl. Table 1: Information on screws used during mechanical testing. All screws and instrumentation were provided by DePuy Synthes.

| System | Diameter outer / inner | Total length | Thread length / pitch | Experiment |
| --- | --- | --- | --- | --- |
| Symphony OCT | 3.5 mm / 2.95 mm | 40 mm | 21 mm / 1.25 | LMS MC |
| Symphony OCT | 3.5 mm / 2.95 mm | 45 mm | 26 mm / 1.25 | LMS BC |
| Symphony OCT | 3.5 mm / 2.95 mm | 40 mm | 31 mm / 1.75 | PS MC |
| Symphony OCT | 3.5 mm / 2.95 mm | 45 mm | 36 mm / 1.75 | PS BC |

LMS – lateral mass screw, PS – pedicle screw, MC – monocortical, BC – bicortical

Suppl.Table 2: Effect of screw positioning (PS or LMS) and fixation (monocortical or bicortical) and sex (male or female) on microstructural/densitometric and mechanical measurements. For this comparison, a two-way ANOVA was performed. The p-value as well as the effect size are reported.

|  |  | Screw/Fixation | | |  | Sex | | |  | Interaction | | |
| --- | --- | --- | --- | --- | --- | --- | --- | --- | --- | --- | --- | --- |
|  |  | *p* |  | $\eta_{p}^{2}$ |  | *p* |  | $\eta_{p}^{2}$ |  | *p* |  | $\eta_{p}^{2}$ |
| BV_total_ (mm^3^) |  | < 0.001 |  | 0.346 |  | 0.003 |  | 0.079 |  | 0.9 |  | 0.002 |
| vBMD (mgHA/mm^3^) |  | < 0.001 |  | 0.191 |  | 0.002 |  | 0.086 |  | 0.9 |  | 0.004 |
| Tt.Length (mm) |  | < 0.001 |  | 0.774 |  | 0.01 |  | 0.061 |  | 0.9 |  | 0.002 |
| Ct.Length (mm) |  | < 0.001 |  | 0.492 |  | 0.3 |  | 0.011 |  | 0.2 |  | 0.042 |
| Tb.Length (mm) |  | < 0.001 |  | 0.511 |  | < 0.001 |  | 0.212 |  | 0.6 |  | 0.016 |
| Loose_Torque_ (Nm) |  | < 0.001 |  | 0.538 |  | 0.1 |  | 0.056 |  | 0.4 |  | 0.059 |
| F_max_ (N) |  | < 0.001 |  | 0.527 |  | 0.2 |  | 0.026 |  | 0.9 |  | 0.011 |
| Displacement_initial_ (mm) |  | < 0.001 |  | 0.483 |  | 0.01 |  | 0.132 |  | 0.7 |  | 0.028 |
| Cycles (1) |  | < 0.001 |  | 0.561 |  | 0.1 |  | 0.05 |  | 0.8 |  | 0.018 |
| Stiffness_initial_ (N/mm) |  | < 0.001 |  | 0.534 |  | 0.003 |  | 0.165 |  | 0.2 |  | 0.092 |
| Stiffness_end_ (N/mm) |  | < 0.001 |  | 0.547 |  | 0.2 |  | 0.032 |  | 0.9 |  | 0.011 |

BV_total_: total bone volume, vBMD: apparent volumetric bone mineral density, Tt.Length: total insertion length, Ct.Length: cortical contact length, Tb.Length: estimated trabecular contact length, F_max_: maximal force, Stiffness_inital_: stiffness at cycle no. 1, Stiffness_end_: stiffness at end of testing.
